# Metal Organic Framework Micro/Nanopillars of Cu(BTC)·3H_2_O and Zn(ADC)·DMSO

**DOI:** 10.3390/nano5020565

**Published:** 2015-04-09

**Authors:** Arben Kojtari, Hai-Feng Ji

**Affiliations:** Department of Chemistry, Drexel University, Philadelphia, PA 19104, USA; E-Mail: ak865@drexel.edu

**Keywords:** nanopillars, micropillars, nanowire, metal-organic frameworks (MOFs)

## Abstract

In this work, we report the optical and thermal properties of Cu(BTC)·3H_2_O (BTC = 1,3,5-benzenetricarboxylic acid) and Zn(ADC)·DMSO (ADC = 9,10-anthracenedicarboxylic acid, DMSO = dimethyl sulfoxide) metal-organic frameworks (MOFs) micro/nanopillars. The morphologies of MOFs on surfaces are most in the form of micro/nanopillars that were vertically oriented on the surface. The size and morphology of the pillars depend on the evaporation time, concentration, solvent, substrate, and starting volume of solutions. The crystal structures of the nanopillars and micropillars are the same, confirmed by powder XRD. Zn(ADC)·DMSO pillars have a strong blue fluorescence. Most of ADC in the pillars are in the form of monomers, which is different from ADC in the solid powder.

## 1. Introduction

Metal-organic frameworks (MOFs), a porous, three-dimensionally linked coordination network materials [1,2] have been used for storage, purification, and separations of gases [3,4] as well as for drug release/delivery [5] heterogeneous catalysis [6,7] and sensing [8]. Due to their unique inorganic–organic hybrid nature and nanometer porous structures, MOFs are rich in fundamental properties which promise revolutionary new device concepts. Most of the studies in the past focused on the rational design, synthesis, characterization, and applications of MOFs in their micro-sized cubic crystals obtained from traditional MOF synthesis reaction. Recently, 2D coatings of MOFs and 1D micro/nanostructures, and MOF-based devices are gaining interests because they can be processed into integrated devices. The 2D coatings include polycrystalline thin films [9,10], SURMOF crystalline nanofilms [11], MOF-coated silicon nanowires [12], patterned growth [13], and single crystal arrays [14]. 1D nanowire structures of MOFs have also emerged [15], but a key barrier to wide-scale integration of functional 1D nanostructures into devices is the difficulty of reproducibly forming programmed contacts between nanowires and substrates.

Recently, we developed a surface-assisted approach for mass-production of 1D MOF micro/nanowire arrays that are vertically oriented on substrates [16]. Research in vertically oriented micro/nanowires, which are also called micro/nanopillar [17], is a rapidly growing area in the last decade because of the unique orientation of pillars and their easy-of-use for wide applications, such as photonic devices, cell growth and imaging [18], antireflection [19], light trap [20], battery [21], laser [22], photodetector [23], photovoltaics [24], light-emitting diodes [25], surface-enhance Raman spectroscopy (SERS) signal enhancing [26], drug delivery [27], sensors [28], and enhanced selective catalysis [29]. The uniqueness and advantage of the micro/nanopillar is that one end of each micro/nanopillar is mechanically connected to the surface when the micro/nanopillars are fabricated. In our preliminary work [30], we reported the fabrication procedure and single crystal structures of the first examples of the MOF micro/nanopillars on surfaces. In this work, we report the characterizations of these pillars with powder crystal diffraction pattern, thermal analysis, and fluorescence of the pillars.

## 2. Results and Discussion

### 2.1. SEM Images of the Nanopillars

**Figure 1:**
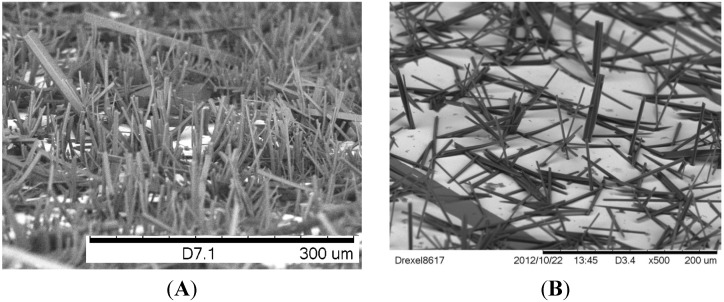
Scanning electron micrographs (SEM) images of (**A**) dense Cu(BTC)·3H_2_O nanopillar array and (**B**) dense Zn(ADC)·DMSO nanopillars grown on gold substrates. Samples were prepared by mixing a saturated BTC in water or ADC in DMSO solutions along with 30 mM CuSO_4_·5H_2_O or 30 mM Zn(NO_3_)_2_·6H_2_O, respectively, in equal volume quantities.

Figure 1 shows the Scanning electron micrographs (SEM) images of Cu(BTC)·3H_2_O and Zn(ADC)·DMSO micro/nanopillar arrays on gold surfaces of substrates. The fabrication procedure has been reported in our previous work [30].

In general, the arrays were prepared by immersing gold coated silicon substrates in solutions that are used for synthesizing MOFs. MOFs form in both solutions and on the surfaces. The morphologies of MOFs on surfaces are most in the form of micro/nanopillars that were vertically oriented on the surface. Typical lengths ranged from 10 to 40 µm and diameters in the nanometer range. As evaporation time is extended to weeks both the diameter and the length of the micropillars increase. A similar result can be achieved by increasing the starting volume of both metal ion and ligand solutions used.

Both MOF systems efficiently produced epitaxial growth of micro/nanopillars on gold substrates. Most Cu(BTC)·3H_2_O pillars were oriented perpendicular to the plane of the substrate, but most of the Zn(ADC)·DMSO pillars have variable tilt angles.

### 2.2. Effect of Solvent and Substrate on the Formation of Nanopillars

**Figure 2:**
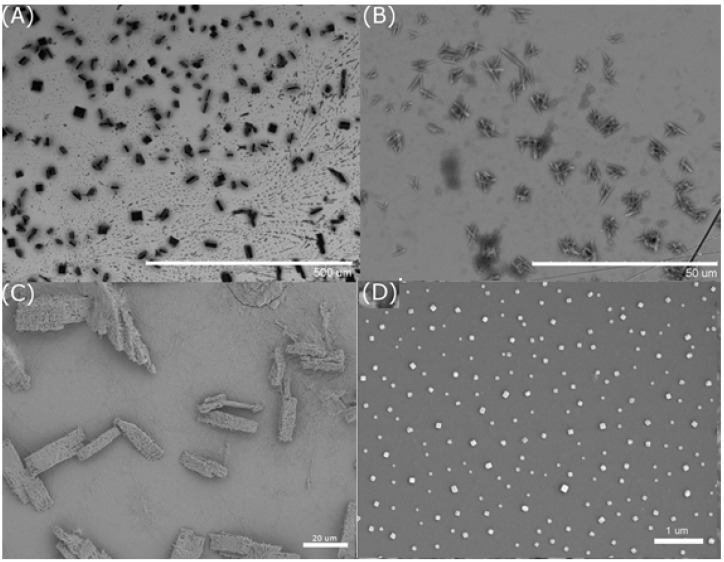
Formation of metal-organic frameworks (MOF) structures of Cu(BTC)·3H_2_O. (**A**) 100 µL of both 30 mM CuSO_4_·5H_2_O and 30 mM BTC in methanolic solutions mixed and completely dried onto gold substrate; (**B**) 0.3 mM of CuSO_4_·5H_2_O and 0.3 mM BTC in methanolic solutions mixed and completely dried onto gold substrate; (**C**) 100 µL of both 30 mM CuSO_4_·5H_2_O and 30 mM BTC in methanolic solutions mixed and completely dried onto silicon substrate; (**D**) 100 µL of both 10 µM CuSO_4_·5H_2_O and 10 µM BTC in aqueous solutions mixed and completely dried onto glass substrate.

Choices of solvent and substrates for both MOF systems were crucial in controlling pillared growth on the substrates. For Cu(BTC)·3H_2_O, methanol was initially used as a solvent to dissolve CuSO_4_·5H_2_O and BTC ligand in millimolar to micromolar concentrations to facilitate crystallization. However, mixing of equivalent amounts of both metal ion and ligand at millimolar concentrations yielded mostly square sheets on substrate surfaces (Figure 2A) while micromolar concentrations resulted in amorphous or mesh structures on gold and silicon substrates (Figure 2B,C). Distilled water supplanted methanol as a solvent choice for CuBTC systems, which yielded cubic structures at 10 µM concentrations on glass or silicon (Figure 2D) and nanopillared systems on gold (Figure 1).

**Figure 3:**
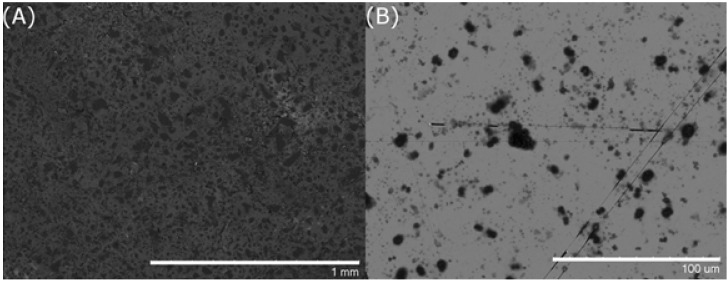
(**A**) 200 µL of 30 mM ADC aqueous solution with 1% NaOH and 30 mM Zn(NO_3_)_2_·6H_2_O aqueous solution mixed and dried completely on gold substrate; (**B**) 200 µL of 0.3 mM ADC aqueous solution with 1% NaOH and 0.3 mM Zn(NO_3_)_2_·6H_2_O aqueous solution mixed and dried completely on gold substrate.

For the ZnADC system, it was first thought to deprotonate both carboxylic acids of the ADC ligand using 1% NaOH to increase the its solubility in distilled water and mixing equimolar and equivalent amounts of both Zn(NO_3_)_2_·6H_2_O and ADC solutions on gold, glass, and silicon substrates. However, precipitation immediately occurs after mixing and non-uniform particles are visible on the surface using SEM with both high and low concentration mixing, which indicates that nucleation did not initiate on the surface but rather in solution (Figure 3A,B). This method was abandoned and a co-solvent system was utilized by dissolving the ADC ligand in DMSO and Zn(NO_3_)_2_·6H_2_O in distilled water. Results showed the growth of nanopillars on gold substrates (Figure 1B), but not on silicon surfaces. Control experiments showed that ADC alone did not form ordered structures in the mixed solvents.

### 2.3. XRD of the Micropillars and Nanopillars

Single-crystal data and refinement parameters for both Cu(BTC)·3H_2_O and Zn(ADC)·DMSO systems are summarized in Table 1.

**Table 1:** Crystal data and structure refinement for the two metal coordination polymers.

Condition and Parameter	Cu(BTC)·3H_2_O	Zn(ADC)·DMSO
Empirical formula	C_9_H_10_O_9_Cu	C_19_H_17_SO_5_Zn
Formula weight	325.71	422.76
Temperature (K)	143(1)	143(1)
Wavelength (Å)	0.71073	0.71073
Crystal system	monoclinic	orthorhombic
Space group	P2_1_/*n*	Pnma
*a* (Å)	6.7778(7)	7.3053(7)
*b* (Å)	18.8206(18)	17.5056(14)
*c* (Å)	8.5384(8)	12.6731(12)
β (°)	92.471(4)	-
Volume (Å^3^)	1088.16(18)	1620.7(3)		
*Z*	4	4		
Density (calculated, Mg/m^3^)	1.988	1.733		
µ (mm-1)	2.052	1.674		
*F*(0 0 0)	660	868		
Crystal size	0.30 × 0.22 × 0.12 mm^3^	0.25 × 0.02 × 0.01 mm^3^		
θ range (°)	2.16 to 27.53	1.98 to 27.53		
Index ranges	−8 ≤ *h* ≤ 8, −24 ≤ *k* ≤ 24, −11 ≤ l ≤ 11	−9 ≤ *h* ≤ 9, −22 ≤ *k* ≤ 22, −15 ≤ l ≤ 16		
Reflections collected	37602	22496		
Independent reflections	2488 (*R*(int) = 0.0170)	1929 (*R*(int) = 0.0557)		
Completeness to θ = 27.53°	99.7%	99.9%		
Absorption correction	Semi-empirical from equivalents	Semi-empirical from equivalents		
Max. and min. transmission	0.7456 and 0.6410	0.7456 and 0.6511		
Refinement method	Full-matrix least-squares on *F*^2^	Full-matrix least-squares on *F*^2^		
Data/restraints/parameters	2488/0/173	1929/108/128		
Goodness-of-fit on *F*^2^	1.112	1.090		
Final *R* indices (I > 2sigma(I))	*R*1 = 0.0194, w*R*2 = 0.0526	*R*1 = 0.0366, w*R*2 = 0.0894		
*R* indices (all data)	*R*1 = 0.0196, w*R*2 = 0.0526	*R*1 = 0.0578, w*R*2 = 0.0996		
Largest diff. peak and hole	0.442 and −0.383 e·Å^−3^	0.970 and −0.660 e·Å^−3^		

**Figure 4:**
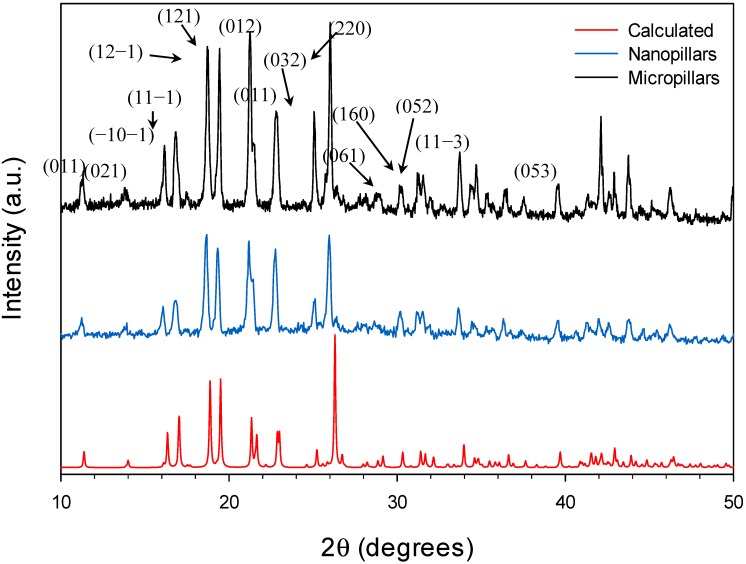
XRD diffraction patterns of Cu(BTC)·3H_2_O micropillars and nanopillars, compared to the calculated pattern obtained via Cambridge Crystallographic Data Center (CCDC).

The single crystal XRD study revealed the crystallographic structure of the Cu(BTC)·3H_2_O micropillars. However, the crystals of nanopillars are too small to be determined by using single crystal XRD. In order to confirm the structure and crystallinity of the fabricated nanopillars, we performed powder XRD to compare the diffraction patterns of the micropillars and nanopillars, as well as to the calculated pattern of Cu(BTC)·3H_2_O. For these experiments, both micro- and nanopillars were prepared in bulk and carefully removed from the gold surfaces for the analyses. Our powder XRD pattern results show strong similarities between the two experimental conditions described in this paper (Figure 4). In addition, the diffractograms for both crystal sizes are agreeable with the calculated pattern using the structure previously published [16].

### 2.4. Thermal Analysis of the MOF Pillars

**Figure 5:**
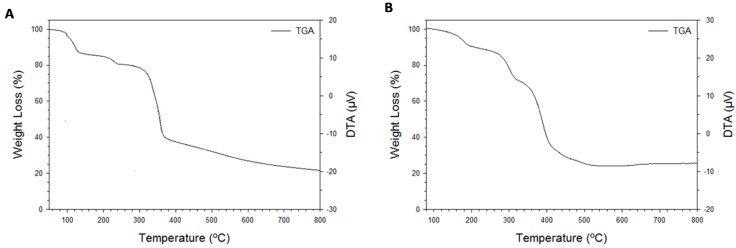
Thermal gravimetric (TG) analyses of (**A**) Cu(HBTC)·3H_2_O and (**B**) Zn(ADC)·DMSO crystals harvested from gold surfaces after pillared growth.

The thermal properties of the metal-coordination polymer nanopillars are shown in Figure 5. Three weight losses were observed in the thermalgravimetric analysis of Cu(BTC)·3H_2_O crystals (Figure 5A). The onset of the first weight loss occurs at 75 °C, which can be attributed to the release of two out of the three Cu^2+^ coordinated water molecules. The loss of the third coordinated water molecule within the structure is removed with an observed weight loss at 205 °C. The observed total weight loss of all three coordinated water molecules is 17.2%, which is agreeable with the calculated percent of water weight loss of 16.5%. The 62.7% weight loss observed at the onset of 270 °C corresponds to the loss of the BTC ligand, which is close to the calculated value of 64.1%. The remaining mass can be attributed to copper metal (calculated 19.4%) and the degraded sample, with an observed percent mass of 20.1% at 800 °C. The weight losses observed correspond to a 3:1:1 ratio between the water molecules, copper metal, and the BTC ligand, respectively. These results are highly agreeable with the crystal structure of the network.

To understand thermal stability of the crystals for future gas absorption studies, thermal analysis was studied. For the Zn(ADC)·DMSO crystals, the thermalgravimetric analysis showed three weight losses (Figure 5B). The onset of the first mass loss was observed at 120 °C, which would correspond to the loss of DMSO solvent absorbed within the pores of the crystals. The mass loss associated with the loss of absorbed DMSO was found to be approximately 10.0%. The onset of the second loss in mass occurred shortly afterwards with a total mass loss of 14.9%. It is thought that is associated with the loss of DMSO coordinated to Zn^2+^ within the coordination network. The onset of the third mass loss occurs at 310 °C, which corresponds to the loss of the ADC ligand (50.4%). The remaining 24.7% mass is mostly due to Zinc metal and degraded carbon in the sample. The calculated ratio of 1:1 for DMSO and ADC, respectively, is agreeable with the crystal structure.

### 2.5. Fluorescence of Zn(ADC)·DMSO Pillars

**Figure 6:**
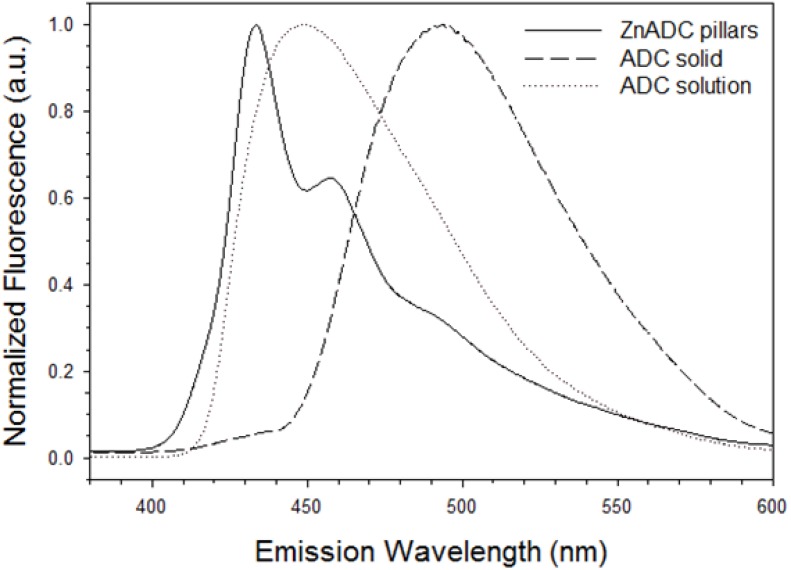
Fluorescence emission spectra of Zn(ADC)·DMSO and 9,10-anthracenedicarboxylic acid using solid-state and solution (λ_ex_ = 320 nm).

Zn(ADC)·DMSO pillars have a strong blue fluorescence. Figure 6 shows fluorescence spectra (λ_ex_ = 320 nm) of Zn(ADC)·DMSO micro/nanopillars, ADC powder, and a 30 mM DMSO solution of ADC. The ADC solid exhibited a far red-shift with an emission maximum at 494 nm, compared to the maxima of the ADC DMSO solution at 448 nm. The peak in the solution is from the ADC monomer. The emission profiles observed in the powder are attributed to the excimer of ADC, which is common for solid state of polyaromatic hydrocarbons [30,31,32]. This 46 nm red-shift from solution to the powder state is attributed to J-aggregation of the anthracene molecules due to π–π stacking [31].

The emission peaks of Zn(ADC)·DMSO micro/nanowires are at 434 nm and 457 nm. These peaks also belong to the monomer of ADC [30]; however, with a slight blue-shift. The blue shift is more often due an increase in energy between S_0_ and S_1_ energy states, which likely arises from the change of the carbonyl resonance by the Zn^2+^ ion [33]. This result is consistent with the observation in XRD, *i.e.*, no π–π interaction between ADC molecules in ZnADC nanopillars. The aforementioned luminescence results were expected for a MOF system using a fluorescent PAH-based ligand.

**Figure 7:**
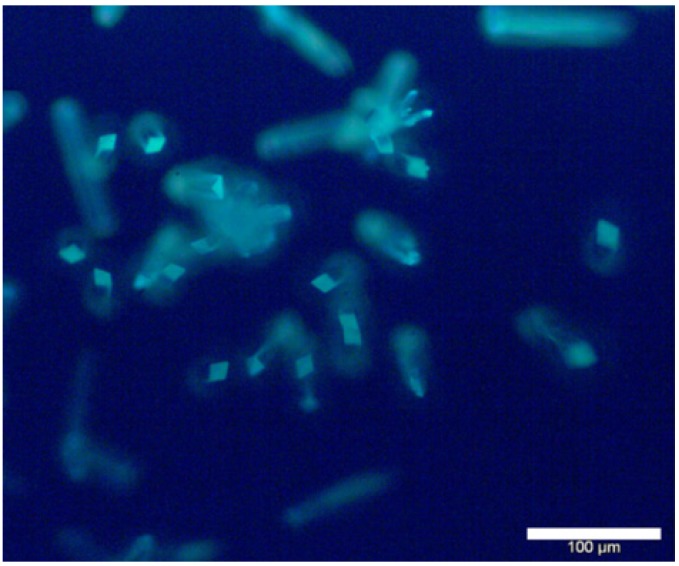
Fluorescence microscope image of Zn(ADC)·DMSO micro/nanopillars.

Figure 7 shows a fluorescence microscope image of Zn(ADC)·DMSO micro/nanopillars. Due to the light propagation along the long direction of nano/microwires, the fluorescence of the Zn/ADC nano/microwires is significantly brighter at the end of the wires, which is the outcoupling light resulting from highly oriented molecular arrangement of the crystal [34], suggesting the Zn/ADC nano/micropillars may be used as a waveguide material for optical applications. The MOF nanopillars prepared in this work can be made in large quantities at a low cost due to the facile self-assembling method.

## 3. Experimental Section

CuSO_4_·5H_2_O (Fisher Scientific), Zn(NO_3_)_2_·6H_2_O (Baker Chemical), 1,3,5-benzenetricarboxylic acid (BTC, Alfa Aesar, Ward Hill, MA, USA), 9,10-anthracenedicarboxylic acid (ADC, Sigma Aldrich, St. Louis, MO, USA), and solvents methanol and dimethyl sulfoxide (Reagent/ACS grade, Pharmco-AAPER, Brookfield, CT, USA) were used as received. Thin-film gold substrates were prepared by e-beam evaporation (BOC Edwards, Crawley, UK). 150 mm-diameter test silicon wafers (Wacker Silicone, Munich, Germany) were coated with a 3 nm thin film of chromium, followed by a 20 nm-thick deposition of gold. Prior to use, substrates were gently rinsed with deionized water and methanol, followed by treatment with UV/ozone (Bioforce ProCleaner, Ames, IA, USA) for 20 min.

Solid state and solution fluorescence spectra were obtained by using a Hitachi F-7000 fluorescence spectrophotometer using an excitation wavelength (λ_ex_) of 320 nm with both the excitation and emission slit width at 5.0 nm. Fluorescence and optical microscopy images were taken using an Olympus BX51 microscope with an Olympus U-RFL-T Mercury burner. ATR infrared spectra were recorded using a Smiths IlluminatIR microscope. Thermal analyses were conducted using a thermogravimeter/differential thermal analyzer (TG/DTA 6300, SII Nanotechnology, Tokyo, Japan) using a heating rate of 10 °C·min^−1^ with a regulated nitrogen gas flow of 50 cm^3^·min^−1^. Scanning electron micrographs (SEM) were obtained using a Hitachi Tabletop Microscope (TM-1000) and Zeiss Supra 50VP with Oxford energy-dispersive X-ray detection (EDX). Micrographs obtained using the Zeiss Supra 50VP were obtained using the SE2 Inlens. Crystallographic data was collected using a Bruker Kappa APEX II Duo CCD diffractometer. Structure solution and refinement was performed using Bruker’s SHELXTL package. Powder X-ray diffraction (XRD) patterns were obtained using a Rigaku Smartlab diffractometer using CuKα_1_ radiation in Bragg-Brentano focusing and Debye-Scherrer geometry. Calculated XRD patterns were obtained using the Mercury crystal visualization software package. Crystal structure files were obtained from the Cambridge Crystallographic Data Center (CCDC).

## 4. Conclusions

Crystalline micro/nanowires of Cu(BTC)·3H_2_O and Zn(ADC)·DMSO were characterized in this work. The facile approach for micro/nanopillar synthesis allows rapid pillared growth with high crystallinity of the MOF systems presented. The MOF micro/nanopillars may have controllable interconnection of the electronic and optoelectronic devices through a vertical integration process using no lithography. This process is low cost and can be high throughput, and offers significant advantages over other methods to prepare nanowire based devices.
